# Aggregate-Depleted Brain Fails to Induce Aβ Deposition in a Mouse Model of Alzheimer's Disease

**DOI:** 10.1371/journal.pone.0089014

**Published:** 2014-02-12

**Authors:** Claudia Duran-Aniotz, Rodrigo Morales, Ines Moreno-Gonzalez, Ping Ping Hu, Joseph Fedynyshyn, Claudio Soto

**Affiliations:** 1 Mitchell Center for Alzheimer's Disease and Related Brain Disorders, Department of Neurology, University of Texas Houston Medical School, Houston, Texas, United States of America; 2 Universidad de los Andes, Facultad de Medicina, Santiago, Chile; 3 Education Ministry Key Laboratory on Luminescence and Real-Time Analysis, College of Life Sciences, Southwest University, Chongqing, China; 4 Novartis Vaccines and Diagnostics, Emeryville, California, United States of America; Creighton University, United States of America

## Abstract

Recent studies in animal models of Alzheimer's disease (AD) show that amyloid-beta (Aβ) misfolding can be transmissible; however, the mechanisms by which this process occurs have not been fully explored. The goal of this study was to analyze whether depletion of aggregates from an AD brain suppresses its *in vivo* “seeding” capability. Removal of aggregates was performed by using the Aggregate Specific Reagent 1 (ASR1) compound which has been previously described to specifically bind misfolded species. Our results show that pre-treatment with ASR1-coupled magnetic beads reduces the *in vivo* misfolding inducing capability of an AD brain extract. These findings shed light respect to the active principle responsible for the prion-like spreading of Alzheimer's amyloid pathology and open the possibility of using seeds-capturing reagents as a promising target for AD treatment.

## Introduction

Misfolding and aggregation of amyloid-β (Aβ) is one of the central pathological features of Alzheimer's disease (AD) [1]–[4]. This process has been linked to cell toxicity, synaptic impairment and brain inflammation [3], [5]. All these deleterious events are associated to tissue dysfunction, leading to clinical signs involving mostly memory loss [6].

Recent experiments have shown that Aβ and other disease-associated proteins can transmit misfolding in a similar way as infectious prions propagate in Transmissible Spongiform Encephalopaties (TSEs) [7]–[10]. Despite the low incidence of prion diseases among human population, the unorthodox nature of the infectious agent [11], the potential of pre-symptomatic individuals to spread disease [12], and the risk of zoonotic transmission [13], have raised significant concerns in terms of basic science and public health. Although no clinical or epidemiological studies suggest inter-individual transmission of Aβ pathology, it is important to fully understand the potential contribution of this recently recognized process in the disease progression.

Prion-like propagation not only represents a risk of transmission among individuals, but may also explain the spreading of misfolded particles among different cells and tissues [14]–[16]. Furthermore, interventions aimed to capture misfolded aggregates while in transit between cells and tissues may constitute a novel and more amenable strategy to treat these devastating diseases. For this reason, the development of tools able to specifically capture and isolate misfolded protein aggregates seems to be crucial in order to design effective diagnostic and therapeutic strategies. It has been recently described that the Aggregate Specific Reagent 1 (ASR1) molecule (a peptoid mimicking the 23–30 aminoacidic sequence of the human prion protein) is able to specifically bind misfolded protein aggregates associated with different pathologies such as Type-2 diabetes, Parkinson's disease, AD, TSEs, among others [17]. Due to its particular properties, the ASR1 compound has been proposed as a promising and sensitive tool to diagnose Protein Misfolding Disorders (PMDs).

In this study, we tested the ASR1 compound as a putative agent to bind and remove misfolded species from an AD brain sample and its effect on the acceleration of cerebral accumulation of amyloid deposits in a transgenic mouse model of AD. Our results show that ASR1-depletion of Aβ aggregates is able to abolish the seeding capability of an AD brain, supporting the future use of this compound for the diagnosis and treatment of AD and other PMDs.

## Materials and Methods

### Transgenic mice

APP_Swe_/PSEN1_ΔE9_ transgenic mice [18] were used in this study. These mice over-express the human amyloid precursor protein harboring the Swedish mutation (K670M and N671L) and a mutant version of the human presenilin 1 gene (PSEN1_ΔE9_). As a result, these animals develop amyloid plaques starting at 4–5 months old [19]. Animals were housed in groups of up to 5 in individually ventilated cages under standard conditions (22°C, 12 h light–dark cycle) receiving food and water *ad libitum*. All animal manipulations were carried out in accordance to NIH regulations and approved by the Animal Welfare Committee of the University of Texas Medical School at Houston. Surgical procedures were performed under isofluorane anesthesia and all efforts were made in order to minimize suffering. 5–8 animals per group were used for this study.

### Human samples, inocula preparation, and injection

Frozen and paraffin fixed temporal cortex specimens from AD (81 years old, male) and non-demented (59 years old, male) individuals were supplied by the National Disease Research Interchange (Philadelphia, PA, USA). Age difference between control and AD samples were due to the lack of available age-matched controls not displaying any amyloid pathology in the brain. It is well-known that cognitively normal people over the age of 60 years old show scattered presence of Aβ aggregates [20]–[22]. We recently reported that intra-cerebral injection of brain extracts from subjects affected by mild cognitive impairment and even non-demented people harboring abundant amyloid pathology are able to accelerate amyloid deposition in AD-transgenic mice [23]. Selection of the appropriate control was performed after an exhaustive histopathological screening. The control sample utilized for this experiment showed no presence of amyloid lesions in several brain areas. 10% w/v brain extracts were prepared with frozen tissues in cold-PBS supplemented with a cocktail of proteinase inhibitors (Roche Diagnostics GmbH, Mannheim, Germany). Removal of aggregated Aβ species was performed by using the ASR1 compound [17] bound to magnetic beads. Briefly, 1 volume of tissue homogenate was mixed with 1 volume of ASR1-bead suspension (30 mg/mL) and incubated in an Eppendorf thermomixer (Hamburg, Germany) at 37°C and 850 rpm for 1 h. Thereafter, beads were immobilized in a magnetic separator and supernatant was collected. Supernatant was submitted to two additional ASR1 treatments and the final samples were stored at −80°C until used for *in vivo* and *in vitro* analyses. The same process was conducted for a different aliquot of the same brain homogenate using magnetic beads lacking the ASR1 peptoid. 30 days-old anesthetized APP_Swe_/PSEN1_ΔE9_ mice were intra-cerebrally injected with 10 µL of treated or untreated brain homogenates into the right hippocampus using the following coordinates: anterioposterior (AP): −1.8 mm; mediolateral (ML): −1.8 mm; dorsoventral (DV): −1.8 mm. Challenged mice and untreated controls were sacrificed by CO_2_ inhalation at 6 months old. Brains were removed and cut in half by the sagittal plane. The right hemisphere was post-fixed into fixative solution (10% neutral buffered formalin) and embedded in paraffin, while the left hippocampus was dissected out, snap-frozen in liquid nitrogen, and stored at −80°C until use. Written consent was obtained to use human derived samples. Handling of human samples was conducted as directed by the Institutional Review Board and Biological Safety Office of The University of Texas Medical School at Houston.

### Immunohistochemistry and Fluorescent staining

10-µm-thick serial sections (one every ten contiguous slides starting from 0.4–0.6 mm lateral) from all animal groups (n = 5–8/group; 5 sections/stain/animal) were processed in parallel for immunostaining. Sections were deparaffinazed and the endogenous peroxidase activity was blocked with 3% H_2_O_2_/10% methanol in PBS for 20 min. Sections were treated with formic acid 85% for 5 minutes for antigen retrieval. After rinsing, the sections were incubated overnight at room temperature with the 4G8 anti-Aβ monoclonal antibody (1∶1,000 Covance, Princeton, NJ, USA. Epitope: VFFAE corresponding to positions 17–24 of Aβ). Slices were later incubated for 1 hour in HRP-linked sheep anti-mouse antibody (1∶1,000 GE Healthcare, Little Chalfont, UK). Peroxidase reaction was visualized using DAB Kit (Vector) following the manufacturer's instructions. Finally, sections were dehydrated in graded ethanol, cleared in xylene, and cover slipped with DPX mounting medium (Innogenex, San Ramon, CA, USA). For Thioflavin-S staining, tissue slices were incubated in ThS (Sigma-Aldrich, St. Louis, MO, USA) solution (0.025% in 50% ethanol) for 5–10 min after deparaffinization. Sections were dehydrated in graded ethanol, cleared in xylene, and cover slipped with DPX mounting medium. Staining of human AD sample was performed using a similar procedure. Anti-hyperphosphorylated tau immunostaining was performed using the AT8 antibody (which recognizes phosphorylated serine 202 and threonine 205; Pierce, Rockford, IL, USA) at a 1∶100 dilution. After incubation with HRP-linked sheep anti-mouse antibody, human samples were developed with DAB and counterstained with hematoxylin.

### Quantification of Anti-Aβ and ThS staining

Sections were examined under a bright field/epifluorescent DMI6000B microscope (Leica, Wetzlar, Germany) and image analysis quantifications were performed using the ImagePro software (Rockville, MD, USA). Burden was defined as the area of the brain stained with anti-Aβ antibodies or ThS per total area analyzed. Burden quantification was performed by an investigator blind to the experimental groups. Lateromedial sagittal slides from all animal groups (n = 5–8 mice per group) were quantified in every tenth sections, in a total of 5 sections.

### Aβ quantification by ELISA

Mice hippocampi were homogenized at 10% w/v in PBS plus proteinase inhibitors. Resulting extracts were fractioned by using a previously described serial extraction protocol [24] with minor modifications. Briefly, 200 µL of tissue homogenates were ultracentrifuged at 32,600 rpm for 1 h at 4°C in a 42.2 Ti rotor (Beckman-Coulter, Brea, CA, USA). Supernatants were collected and pellets were resuspended in 200 µL of 70% formic acid (Fisher Scientific, Waltham, MA, USA) by pipetting and sonication. Samples were centrifuged for 30 min at the same conditions described above and supernatants were collected. Formic acid fractions were diluted 20 times in 1 M Tris buffer, pH 11 (Sigma-Aldrich, St. Louis, MO, USA) in order to neutralize pH. Human brain homogenates were processed in the same way as described above, but one additional SDS extraction step (by resuspending the pellets obtained after the first centrifugation step in 2% SDS prepared in PBS) was performed. SDS supernatants were diluted 40 times in EC buffer (0.02 M phosphate buffer, pH 7, 0.4 M NaCl, 2 mM bovine serum albumin, 0.05% CHAPS and 0.05% sodium azide) and pellets were resuspended in 70% formic acid to resume the extraction process. Brain levels of Aβ_42_ and Aβ_40_ were measured using Human Aβ ELISA Kits (Invitrogen, Carlsbad, CA, USA). Samples were read on an ELISA plate reader (EL800 BIO-TEK, BioTek, Winooski, VT, USA) at 450 nm.

### Total protein evaluation by Coomasie staining and BCA assay

For Coomasie staining, 20 µL of PBS, SDS and formic acid fractions (diluted as previously described) were separated by SDS-PAGE using 4–12% gels (Invitrogen, Carlsbad, CA, USA). Gels were cleared with water and stained in Coomasie solution (3 g/L brilliant blue, 50% methanol, and 10% acetic acid) for ∼2–3 h with gentle shaking at room temperature. Later, gels were washed in washing buffer (50% water, 40% methanol and 10% acetic acid) overnight at 4°C. Next day, gels were washed again for ∼2–3 h at room temperature and pictures were taken. Bicinchoninic acid (BCA) assay was performed in the same samples used for Coomasie staining as recommended by the manufacturer (Pierce, Rockford, IL, USA).

### Statistical analysis

Data were expressed as means ± standard error (SEM). After confirming normal distribution with Skewness and Kurtosis statistic test, one way analysis of variance (ANOVA) followed by a post-hoc Tukey's multiple comparisons test was used to analyze differences among groups. Statistical analyses were performed using GraphPad Prism 5.0 software (GraphPad Software Inc). Statistical differences for all tests were considered significant at the p<0.05 level.

## Results

### ASR1 removes Aβ aggregates from an AD human brain extract

The prion-like properties of misfolded Aβ have been extensively studied in mice models of AD [7], [8], [25]. Direct intra-cerebral injections of ASR1-treated and untreated AD brain homogenates into transgenic mice models of AD were done to determine whether ASR1 can ameliorate the misfolding transmitting features of aggregates present in these samples. Before animal bioassays, the AD human brain to be used as inoculum in our studies was characterized. For this purpose, the profile of Aβ and tau deposition in the temporal cortex was analyzed (Figure S1). Formalin-fixed samples were stained using the anti-Aβ 4G8 antibody and thioflavin-S (ThS) to detect Aβ deposition and fibrillar aggregates, respectively. Additionally, the AT8 antibody was used to detect hyper-phosphorylated tau. As shown in Figure S1A, the AD brain sample displayed a typical pattern of Aβ deposition, including mature plaques (open arrows) and diffuse aggregates (solid arrows). Moreover, the presence of tangles in this sample was clearly observed (arrow heads). Furthermore, the Aβ content in the AD sample was compared to the one observed in the brain of a non-demented, individual after serial extraction in Phosphate Buffer Saline (PBS), Sodium Dodecyl Sulphate (SDS), and Formic Acid (FA) solutions coupled to ultracentrifugation and anti-Aβ ELISA. As expected, the levels of PBS-insoluble Aβ_40_ (Figure S1B) and Aβ_42_ (Figure S1C) in the AD brain were higher compared to the ones present in the control.

**Figure 1:**
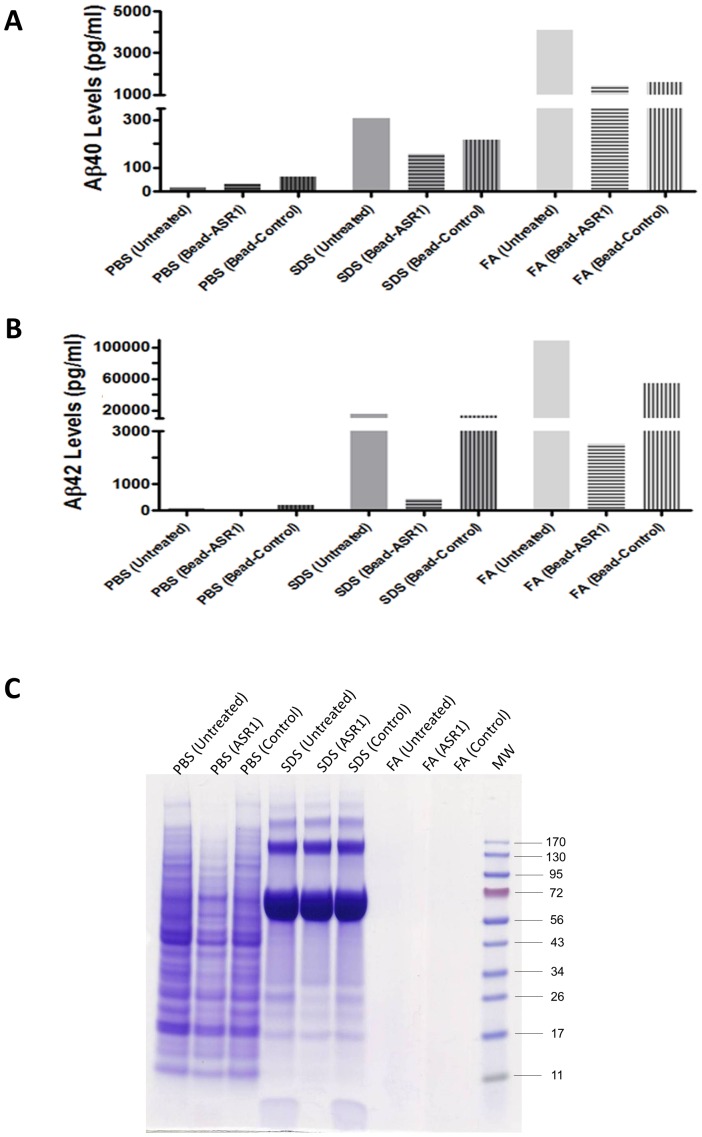
Removal of protein aggregates by ASR1 decreases Aβ levels in AD brain homogenate. Aβ levels of an ASR1-treated AD brain homogenate were biochemically evaluated using anti-human Aβ ELISA kits. The quantity of Aβ_40_ (**A**) and Aβ_42_ (**B**) was measured in different fractions (PBS, SDS, and FA) obtained after serial extraction. Measurements were performed in duplicates and the bars represent the average of the two determinations. Serial extraction was performed in order to assess the content of different aggregated Aβ species (i.e. PBS: monomeric and soluble oligomeric Aβ; SDS: water insoluble oligomers and protofibrils; FA: large fibrilar structures). (**C**) Coomasie staining of SDS-PAGE fractionated PBS, SDS and FA fractions performed to assess total removal of proteins by the ASR1 compound. PBS: Phosphate Buffer Saline; SDS: Sodium Dodecyl Sulphate; FA: Formic Acid; MW: Molecular Weight Marker. Numbers in the right side of (C) represent molecular weight (in KDa).

Removal of protein aggregates from the AD brain extract was performed by using the ASR1 compound bound to magnetic beads (here simply referred as ASR1). Beads lacking the ASR1 compound were used to control the specificity and efficiency of this molecule. The presence of disease-associated Aβ species was assessed by anti-Aβ ELISA using the same procedure explained above (Figure S1B and 1C). ASR1 treatment resulted in a substantial reduction of the PBS-insoluble Aβ levels compared to the control beads and non-treated sample (Figure 1A and B). This reduction was more dramatic for the Aβ_42_ insoluble species. Compared to the untreated AD sample, our results showed that ASR1 was able to reduce ∼20- and ∼35-times the levels of Aβ_42_ in the SDS and FA fractions, respectively. The same sample treated with the control beads showed only a small reduction in both PBS-insoluble Aβ_40_ and Aβ_42_ levels compared to the non-depleted sample. This residual binding was probably due to unspecific binding of Aβ structures, which is not surprising considering the well-known stickiness behavior of multimeric Aβ [26]. We further characterized the effect of the ASR1-associated and control beads on the AD sample by measuring the total protein concentration after treatment. Coomasie-stained samples after SDS-PAGE did not show any apparent reduction of total protein in the PBS and SDS fractions (Figure 1C); however, protein quantification by bicinchoninic acid (BCA) assay showed a reduction of ∼39% in the PBS fraction and only a ∼4% in the SDS fraction in the ASR1 treated sample compared to the non-treated one. A ∼21% of total protein removal on the PBS fraction was caused by the control beads. No signal was detected for the FA fractions in neither of both assays.

### Depletion of Aβ aggregates from AD brain homogenate substantially reduces its ability to accelerate AD pathology *in vivo*

**Figure 2:**
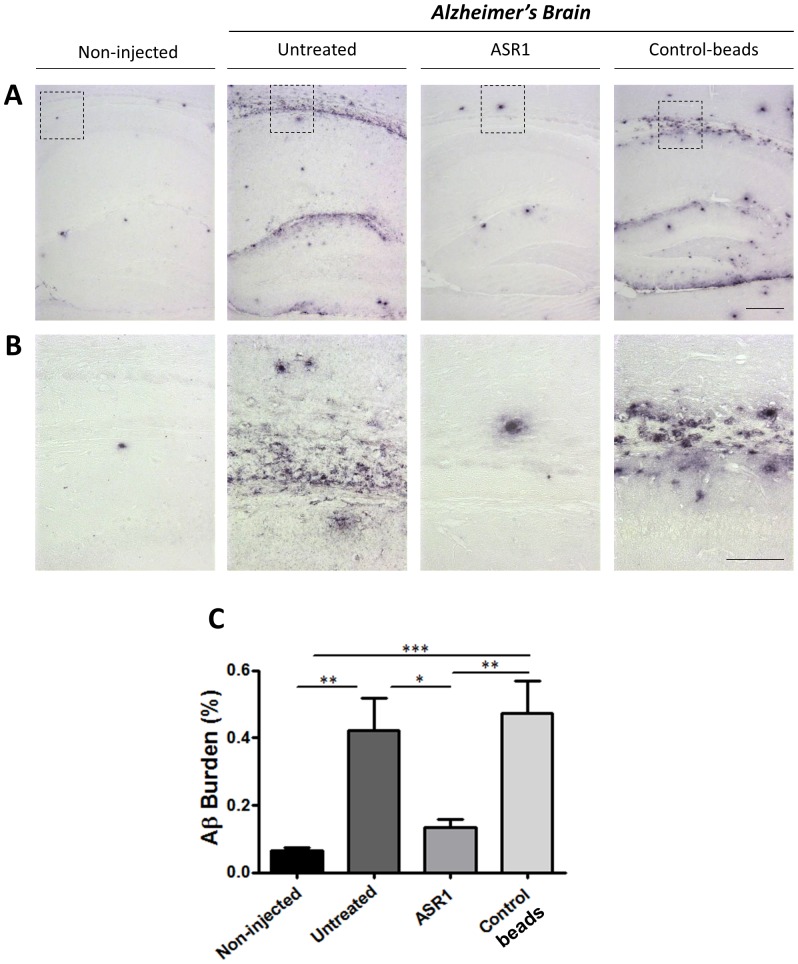
Aggregate-depletion by the ASR1 compound significantly reduces the *in vivo* seeding effect of an AD brain. Brains from experimental and control animals were analysed by immunostaining using the 4G8 antibody. (**A**) Representative pictures of the amyloid deposits found in the hippocampus of non-injected animals, as well as mice injected with AD brain homogenate untreated or treated with ASR1-beads or control-beads. (**B**) Magnification of the area depicted in (A). The scale bar corresponds to 250 µm in (A) and 100 µm in (B). (**C**) Image analysis quantification of the 4G8-stained plaques (burden) in the hippocampus. Aβ burden was defined as the area of the brain labeled with the Aβ antibody per total area analyzed and is expressed as percentage. * P<0.05; ** P<0.01. *** P<0.001.

Brain extracts treated and untreated with ASR1 or control beads were evaluated *in vivo* for their seeding capability in AD transgenic animals. Resulting samples were intra-cerebrally inoculated into 1 month old APP_Swe_/PSEN1_ΔE9_ mice, a transgenic line described to show Aβ aggregates in hippocampus and cortex as early as 4–5 months of age [19]. Injected and non-injected animals were sacrificed at 6 months of age in order to analyze Aβ deposition by histological and biochemical techniques. This time point was carefully selected after characterizing the rate of Aβ deposition in this transgenic line [23]. Endogenous accumulation of amyloid deposits in APP_Swe_/PSEN1_ΔE9_ mice showed an inflexion point between 6–7 months. The time of animal sacrifice was chosen to allow the longest incubation period in which animals will have minimal accumulation of endogenous Aβ aggregates. In our experience, this enables to most clearly differentiate the effect of exogenous seeding from endogenous accumulation of amyloid deposits. We suspect that results obtained at longer incubation periods could be difficult to interpret due to the exponential appearance of amyloid deposits after 7 months of age. Untreated 6 months old mice showed few deposits at this specific age (Figure 2A and B, non-injected), similar to what has been observed for mice injected with PBS or brain homogenates from control patients [23]. On the contrary, a large amount of Aβ deposits in the hippocampal area were found on animals challenged with the untreated AD brain homogenate (Figure 2A and B, untreated). Remarkably, the AD sample treated with the ASR1 reagent was unable to seed Aβ aggregation in the hippocampus (Figure 2, ASR1). The levels of Aβ deposition observed in these animals were not significantly different from non-injected mice (Fig. 2C). Moreover, AD brain treated with control beads resulted in similar levels of Aβ induction when compared with the untreated sample. (Figure 2A and B, Control-beads). Interestingly, a particular pattern of diffuse aggregation was observed in the corpus callosum, hippocampal sulcus and fimbriae of animals injected with the control-beads and untreated AD samples. This feature was absent in the brain of animals from the non-injected and ASR1 treated brain homogenate challenged groups (Figure 2A and B). Quantification of Aβ burden by image analyses further corroborated these findings (Figure 2C).

**Figure 3:**
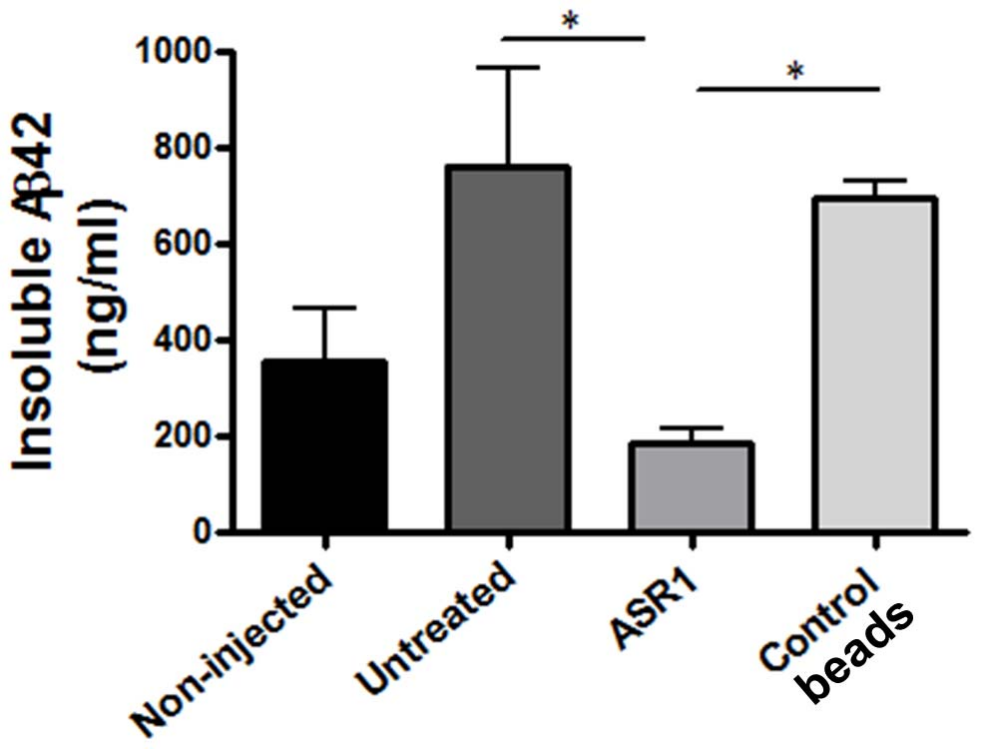
AD-mice injected with aggregate-depleted samples show lower levels of insoluble Aβ42 in their brains. Frozen hippocampi were homogenized and the quantity of aggregated Aβ42 was measured by ELISA after formic acid extraction as explained in the Materials and Methods section. The quantity of insoluble Aβ_42_ was measured using ELISA kits designed to specifically detect this peptide. The values shown correspond to the means ± SEM. * P<0.05.

To further confirm our results, the amount of insoluble Aβ_42_ was measured in the hippocampi of all animals included in this study (Figure 3). In agreement with the results depicted in Figure 2, levels of PBS insoluble Aβ_42_ in ASR1-brain injected animals were similar to the ones observed in the non-injected mice (Figure 3). Aβ_42_ levels in animals injected with the control beads treated sample showed a similar behavior compared to the untreated animals.

**Figure 4:**
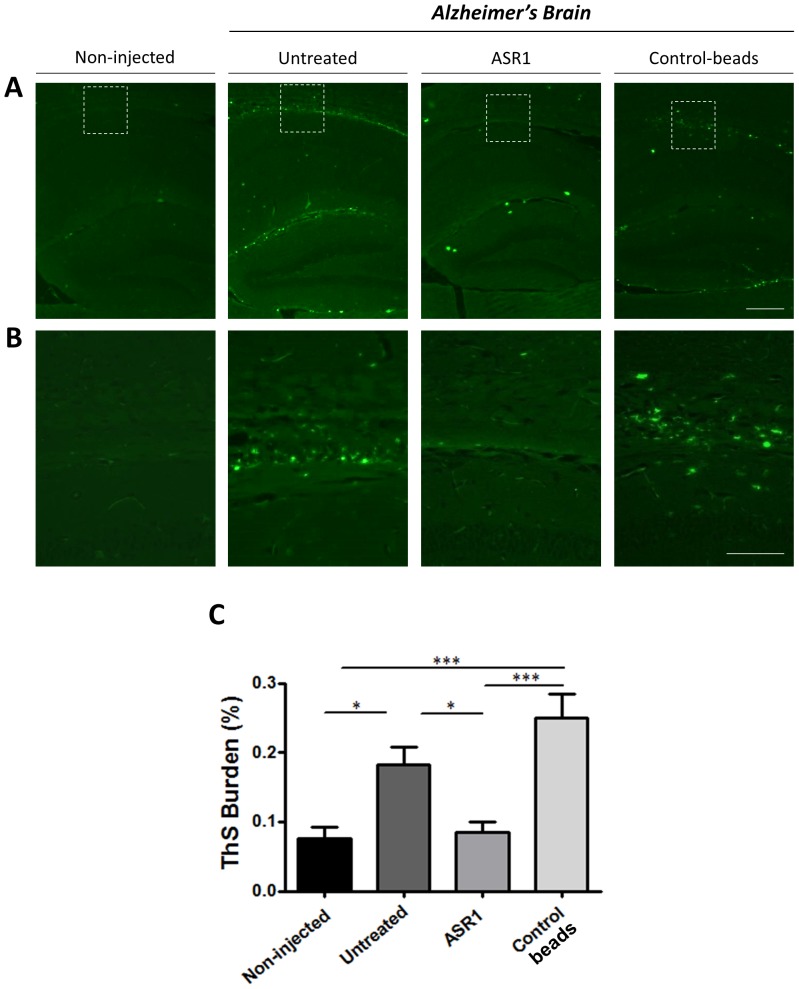
Aggregate removal by ASR1 reduces load of ThS-positive amyloid fibrils. ThS stained brain slices from animals inoculated with different samples were analysed with an epifluorescent microscope. (**A**) Representative pictures of fibrillar Aβ from each group. (**B**) Magnification of the areas marked in (A). The scale bar corresponds to 250 µm in (A) and 100 µm in (B). (**C**) The burden of ThS positive deposits was compared among all different control and experimental groups by image analysis. * P<0.05; *** P<0.001.

Finally, the formation of fibrillar amyloid deposits was evaluated by ThS staining (Figure 4). As shown in Figures 4A and B, fibrillar structures were mostly present in the corpus callosum, hippocampal sulcus and fimbriae, feature that was enhanced in the untreated animals and animals injected with brain homogenate treated with control-beads. As expected, these results were similar compared to those obtained after analyzing brain slices treated with anti-Aβ antibody (Figure 2), confirming that treatment of the brain extracts with the ASR1 compound dramatically reduces the seeding activity of this sample in the hippocampus.

## Discussion

Recent studies have shown that misfolded aggregates from many disease-associated proteins can self-propagate in a process that resembles the replication of yeast and mammalian prions [27]–[29]. These findings have important implications to understand how these structures are formed and accumulate during the disease. However, it remains to be studied whether prion-like spreading and transmission of misfolded aggregates indeed occur under natural conditions in humans. It is likely that, in the same way as in TSEs, the transmission of protein misfolding operates in neurodegenerative diseases much more frequently in the cellular and tissue spreading of misfolded aggregates within an individual, than in the rare cases of inter-individual transmission.

The spreading of misfolded aggregates among cells and tissues also has important implications for treatment. Indeed, capturing aggregates outside cells and even in the peripheral circulation may represent a novel and more reachable approach than attacking a process happening inside brain cells. To accomplish this aim, it is necessary to identify molecules capable to capture specifically misfolded aggregates. These molecules may also be useful in the detection of misfolded aggregates and thus may be utilized for disease diagnosis. Antibodies are a logical option and several of them are currently under development for AD treatment [30]. Another alternative is to engineer molecules to bind with high affinity the misfolded conformation of Aβ aggregates. Several small molecules and amyloid-binding proteins have been shown to bind with high affinity to misfolded Aβ aggregates [31], [32]. Among them, the ASR1 compound has been described to specifically bind the misfolded form of several proteins [17] in a similar way as described for “conformational antibodies” [33], [34]. However, due to its non-peptidic nature, ASR1 has additional advantages in terms of resistance to degradation and clearance.

Taking advantage of the ASR1 ability to bind and capture misfolded aggregates, we studied the effect of incubating an AD brain extract with this molecule on the seeding of amyloid deposition in a mouse model of Aβ amyloidosis. Our results confirmed the efficacy of magnetic beads bound to ASR1 to remove disease-associated Aβ structures (aggregated Aβ_40_ and Aβ_42_). Interestingly, the ASR1 effects were stronger over Aβ_42_, the molecule considered as the most deleterious in this pathology [5]. The specificity of the ASR1 compound over misfolded aggregates was confirmed by the very small reduction in the total amount of proteins observed in all fractions (Figure 1C). Subsequently, we tested the *in vivo* seeding capability of the aggregate-depleted AD samples in APP_Swe_/PSEN1_ΔE9_ mice. By using three independent analysis (anti-Aβ immunostaining, anti-Aβ ELISA, and ThS staining), we observed that the ASR1-treated AD brain homogenate significantly reduced its ability to induce Aβ aggregation in the hippocampus of challenged animals. Conversely, injection of untreated brain extracts and homogenates treated with control-beads resulted in a highly significant induction of Aβ deposition mostly in the corpus callosum and hippocampal fimbriae. Our results indicate that the ASR1 compound can specifically bind biologically active Aβ seeds and inhibit the seeding of these structures in the brain. Since animals were not observed for longer periods of time, we cannot rule out that the reduction of amyloid deposition observed by treating AD-inoculum with ASR1 may not be so evident at later time points. However, reduction of inducible pathology was clear in this specific experimental setup. These findings open the possibility of using seeds-capturing reagents as a promising target for treatment in AD and several other PMDs. Future experiments should address whether this molecule is suitable for *in vivo* administration or would be restricted to *in vitro* pre-treatment of samples designated for human use (i.e. blood, human pituitary extracts, urine, etc.) to deplete them from misfolded aggregated proteins. In order to use ASR-1 for *in vivo* administration, it is necessary to elucidate the efficacy, pharmacokinetics, biodistribution and toxic properties of the molecule. It is also unclear whether ASR1-bound amyloids would be inactivated or stabilized in terms of their seeding effects in an *in vivo* context. This molecule could also be potentially used for basic research by helping in the analysis of common structural motifs present in amyloid assemblies.

## Supporting Information

Figure S1 **Characterization of human Alzheimer's brain inoculum.** (**A**) Pictures show the aggregation profile of Aβ and hyper-phosphorylated tau in the temporal cortex of an AD patient. Open and solid arrows show mature and diffuse plaques, respectively. ThS stained image depicts extracellular and intracellular aggregates, the latter associated to neurofibrillary tangles (right panel, arrow head), as shown in the AT8-stained brain slice. The scale bar corresponds to 100 µm. The quantity of soluble/insoluble Aβ_40_ (**B**) and Aβ_42_ (**C**) was measured by ELISA as described in the text. The values shown are expressed as means ± SEM.
(TIF)
