# Supplementary figures and images for: Aggregate-Depleted Brain Fails to Induce Aβ Deposition in a Mouse Model of Alzheimer's Disease
Source: PLoS One. 2014 Feb 12;9(2):e89014. doi: 10.1371/journal.pone.0089014 (PMC3923072; doi:10.1371/journal.pone.0089014)

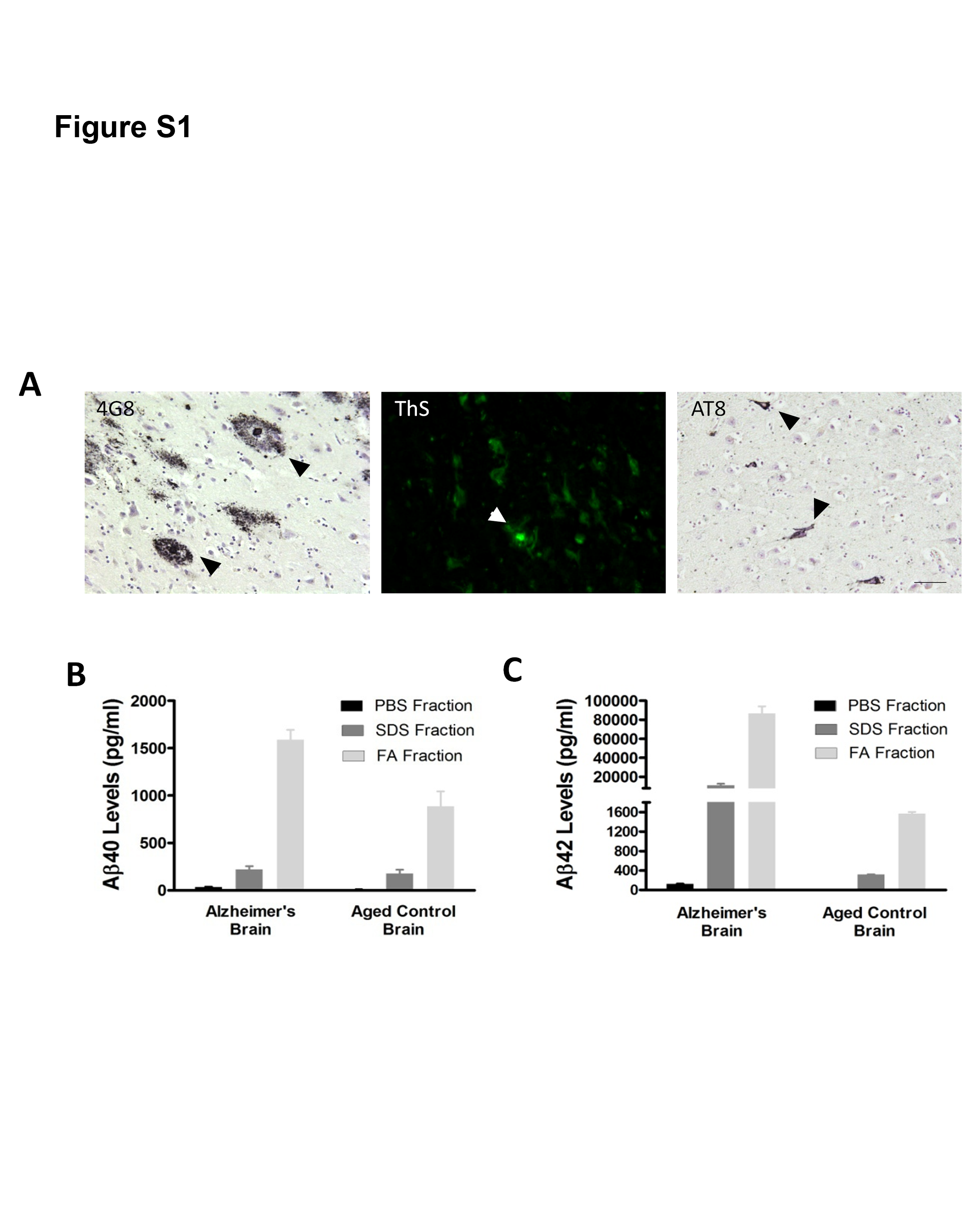

Supplement: Figure S1 — Characterization of human Alzheimer's brain inoculum. (A) Pictures show the aggregation profile of Aβ and hyper-phosphorylated tau in the temporal cortex of an AD patient. Open and solid arrows show mature and diffuse plaques, respectively. ThS stained image depicts extracellular and intracellular aggregates, the latter associated to neurofibrillary tangles (right panel, arrow head), as shown in the AT8-stained brain slice. The scale bar corresponds to 100 µm. The quantity of soluble/insoluble Aβ40 (B) and Aβ42 (C) was measured by ELISA as described in the text. The values shown are expressed as means ± SEM. (TIF) [file pone.0089014.s001.tif]
